# Systemic Disease Associations of Oral Lichenoid Disease: A Retrospective Case–Control Study

**DOI:** 10.1111/odi.70117

**Published:** 2025-10-09

**Authors:** Mari Vehviläinen, Tuomas Selander, Tuula Salo, Maria Siponen

**Affiliations:** ^1^ Department of Oral and Maxillofacial Diseases Faculty of Medicine, University of Helsinki Helsinki Finland; ^2^ Science Service Center Kuopio University Hospital Kuopio Finland; ^3^ Oral Pathology, Research Unit of Population Health, Faculty of Medicine, University of Oulu and Oulu University Hospital Oulu Finland; ^4^ Medical Research Center, Oulu University Hospital Oulu Finland; ^5^ Department of Oral and Maxillofacial Diseases; Translational Immunology Program (TRIMM); iCAN Digital Precision Cancer Medicine Flagship University of Helsinki Helsinki Finland; ^6^ Institute of Dentistry Faculty of Health Sciences, University of Eastern Finland Kuopio Finland; ^7^ Odontology Education Unit and Department of Oral and Maxillofacial Diseases Kuopio University Hospital Kuopio Finland

**Keywords:** allergies, hypothyroidism, oral lichen planus, oral lichenoid lesions, systemic diseases, thyroid diseases

## Abstract

**Objective:**

To analyze the association between oral lichen planus (OLP), oral lichenoid lesions (OLL), and other comorbidities.

**Material and Methods:**

A case–control study of 205 OLP, 96 OLL patients, and 301 age‐ and sex‐matched controls from Kuopio University Hospital was performed. Medical history, regular medications, allergies, lichen planus in the body, and clinical features of OLP/OLL were recorded.

**Results:**

The prevalence of autoimmune hypothyroidism (HT) was 12.2% in OLP, 18.8% in OLL, and 9% in the control group; the estimated odds ratio (OR) was 1.425 (95% confidence interval [CI] 0.793 to 2.562) (*p* = 0.237) for OLP and 2.292 [95% CI 1.175–4.47] (*p* = 0.015) for OLL. Allergies were found in 36.1% of OLP patients and in 22.9% of controls; the estimated OR was 1.872 [95% CI 1.245–2.815] (*p* = 0.003) for OLP. Patients with HT had more often erosive lesions than patients without HT.

**Conclusion:**

The present study suggests that OLL is associated with HT. The association between OLP and HT was not statistically significant. Allergies are associated with OLP.

## 1 Introduction

Oral lichenoid disease encompasses oral lichen planus (OLP) and oral lichenoid lesions (OLL) (Aguirre‐Urizar et al. 2020). While OLP, a mucosal type of lichen planus (LP), is a chronic inflammatory disorder of unknown etiology, OLLs develop in response to an extrinsic agent, such as drugs or dental materials (mainly amalgam), or due to such conditions as graft‐versus‐host disease (Al‐Hashimi et al. 2007). OLP is more common among middle‐aged females and is estimated to occur in 0.11%–3.2% of the adult population (Gonzalez‐Moles et al. 2021; Li et al. 2020). In a study conducted in Northern Finland, the prevalence of oral lichenoid reaction was 2% in a middle‐aged (45–47 years) population (*n* = 1961) and it was more common among males (2.7% vs. 1.3%) (Oivio et al. 2020). OLP has a risk of approximately 1% of malignant transformation (MT), with reported estimates ranging between 0.94% and 1.37% (Aghbari et al. 2017; Giuliani et al. 2019; Gonzalez‐Moles et al. 2019; Li et al. 2023); this risk appears to be higher for OLLs ranging from 1.95% to 2.43% (Giuliani et al. 2019; Li et al. 2023).

Several diagnostic criteria for OLP have been proposed (Cheng et al. 2016; Kramer et al. 1978; van der Meij and van der Waal 2003; Warnakulasuriya et al. 2021). The American Academy of Oral and Maxillofacial Pathology (AAOMP) proposed four clinical criteria for OLP as follows: (1) multifocal symmetric distribution, (2) white and red lesions exhibiting one or more of the following forms: reticular/papular, atrophic (erythematous), erosive (ulcerative), plaque, bullous, (3) lesions are not localized exclusively to the sites of smokeless tobacco placement or adjacent to and in contact with dental restorations, and (4) lesion onset does not correlate with the start of a medication or with the use of cinnamon‐containing products (Cheng et al. 2016). The histopathological criteria for OLP are: (1) band‐like or patchy, predominately lymphocytic infiltrate in the lamina propria confined to the epithelium‐lamina propria interface, (2) basal cell liquefactive (hydropic) degeneration, (3) lymphocytic exocytosis, (4) absence of epithelial dysplasia, and (5) absence of verrucous epithelial architectural change. Unambiguous diagnostic criteria for all OLLs have not been established. Many clinical and histopathological features of OLP can be seen in various OLLs, thus complicating diagnostics. In the AAOMP position paper, disorders that may cause oral lesions resembling OLP include mucous membrane pemphigoid, lichen planus pemphigoides, chronic graft versus host disease (cGVHD), chronic ulcerative stomatitis, oral lichenoid drug reactions (OLDR), oral lichenoid contact hypersensitivity reactions (OLCHR), lupus erythematosus, and proliferative verrucous leukoplakia (Cheng et al. 2016). With anamnestic, clinical, and follow‐up information, diagnostic tests, and clinical and histopathological features, most of these specific disorders can be identified (Cheng et al. 2016). If these can be excluded, the lesions can be classified as OLLs comprising OLDR, OLCHR, and GVHD. In some OLLs, the triggering factor or condition cannot be identified (Xue et al. 2005).

Although the exact pathogenesis of OLP is incompletely understood, a dysregulated T‐cell‐mediated response to either exogenous triggers or thus far unknown autologous keratinocyte antigens is thought to be involved (Kurago 2016). In OLDRs, different routes of antigen presentation appear to be associated with the pathogenesis (McCartan and Lamey 1997). OLCHRs represent a lymphocyte‐mediated delayed type of hypersensitivity reaction (Bolewska and Reibel 1989; Kamath et al. 2015).

Some systemic diseases, such as hepatitis C, thyroid disease, dyslipidaemia, and diabetes, have been associated with OLP (De Porras‐Carrique et al. 2022; Gonzalez‐Moles et al. 2023; Hasan et al. 2019). In two meta‐analyses, a significant association was shown between OLP and thyroid disease, especially hypothyroidism (HT) (De Porras‐Carrique et al. 2022; Li et al. 2017). In addition, a recent Italian study reported that patients with thyroid diseases showed an increased risk of developing OLP (*p* = 0.0007) and OLLs (*p* = 0.0129) (Piloni et al. 2024). A positive association between OLP and HT (OR 2.39) was previously observed also in a Northern Ostrobothnian Finnish population (Siponen et al. 2010). However, significant associations between OLP and HT have not been found in all studied populations (Aghbari et al. 2017; Kats et al. 2019; Lavaee and Majd 2016; Vucicevic Boras et al. 2014).

To date, only a few studies have reported a positive association between OLP and allergies (Siponen et al. 2010; Ting et al. 2023). In a Danish study, no difference in the prevalence of allergies between OLP and control patients was found (Kragelund et al. 2003).

Currently, studies about the association of OLL with systemic diseases, with the exception of GVHD and oral lupus erythematosus (Binnie et al. 2024), are rare. In a previous Finnish study, OLL patients had HT more often than control patients (9% vs. 5%), although the finding did not reach statistical significance (OR 1.75 [95% CI 0.56–4.90]) (Siponen et al. 2010).

The aim of this study was to analyze the systemic disease associations of oral lichenoid disease. In particular, we aimed to elucidate whether the associations of OLP, OLL, thyroid diseases, and allergies previously seen in a Finnish study (Siponen et al. 2010) would be found in a different region of Finland. As most previous studies have excluded patients with OLLs, data on the comorbidities associated with OLL remain limited. Therefore, in addition to examining OLP, the present study aimed to explore potential associations between OLL and systemic conditions.

## 2 Material and Methods

A retrospective case–control study was conducted using data from patients diagnosed with OLP or OLL at Kuopio University Hospital (KUH) between 1996 and 2017, along with age‐ and sex‐matched controls. All cases with histopathological diagnoses of OLP or OLL (*n* = 301) were initially reviewed by the first author (M.V.). Data on the clinical features of the cases (e.g., photographs of the lesions) were retrieved from patient files. The diagnosis of OLP or OLL (excluding specific diseases with OLLs and including OLDR, OLCHR, and GVHD) was determined by applying the classification proposed by the AAOMP in a 2016 position paper (Cheng et al. 2016) as described in the introduction. The histopathological diagnoses were made by an oral pathologist and the clinical and final diagnoses were made by the first author (M.V.).

Patients were excluded if they had a history of head and neck cancer or had received radiotherapy to the head and neck region, due to the established risk of radiation‐induced hypothyroidism. This criterion also encompassed individuals treated with radiotherapy for other tumor types involving the head and neck area, including neck metastases, lymphomas, and central nervous system tumors (Reiners et al. 2020). After this evaluation, the study group consisted of 205 OLP patients and 96 OLL patients.

The control group comprised 301 age‐ (±5 years) and sex‐matched subjects who were randomly and automatically selected from the patients who visited the Oral and Maxillofacial Diseases Clinic of KUH during the same period (1996–2017) as the cases. The most common reasons for the control patients to attend the clinic were specialist‐level orthodontic, prosthodontic, surgical cases (e.g., traumas, cysts), infectious disease, sleep apnea, temporomandibular joint disorder, and pre‐cancer dental treatment. Exclusion criteria comprised a diagnosis of LP, OLP, or OLL, history of head and neck cancer, or radiotherapy to the head and neck region.

Demographic characteristics and medical history (including all medical conditions), medications (thyroxin and other regular medications), history of hepatitis C virus (HCV) infection, allergies (confirmed by a dermatologist to dental materials and self‐reported allergies to different drugs and food allergens), and smoking status were extracted from the medical records of cases and controls by the first author (M.V.). If thyroid gland disease was present, the type was registered. Systemic diseases, medications, and allergies were registered at the time of OLP/OLL diagnosis given at the hospital.

In OLP and OLL patients, a diagnosis of cutaneous or genital LP was recorded if the lesions were diagnosed or suspected by a dermatologist (cutaneous LP) or gynecologist (genital LP) as being LP, diagnosed previously as LP by a physician (according to the patient), or suspected by a hospital dentist to be LP. The localization of cutaneous LP was also recorded.

OLP and OLL patients were divided into three groups based on the clinical type of OLP or OLL. Group 0 included patients with only white lesions (reticular, papular, plaque‐like), group 1 included patients with red, atrophic lesions, with or without white lesions, and group 2 included patients with erosive and bullous lesions, with or without white or red lesions. The patients were grouped by the location of lesions as buccal, gingival, and lingual only, a combination of the former—buccal and gingival, buccal and lingual, several sites (excluding the previously mentioned groups), or other oral site only (palatal mucosa, alveolar ridge mucosa, floor of mouth, labial mucosa, or lip). If the patient had visited the clinic several times, all clinical types and regions of lesions seen at the visits were recorded.

The study protocol was approved by the Ethics Committee of the Northern Ostrobothnian Hospital District (7449/06.01.03.01/2013). Kuopio University Hospital gave organizational consent for the study (215/2017).

Statistical analyses were performed with IBM SPSS Statistics 27 by the first author (M.V.). Descriptive statistics of OLP and OLL characteristics were described by frequencies and percentages or means and ranges. Basic characteristics between groups were compared by analysis of variance and Pearson Chi‐square tests. The associations of OLP, OLL, and OLP/OLL with selected characteristics were examined with a logistic regression model, with age and sex as adjusting variables. Odds ratios (ORs) with 95% confidence intervals (CIs) were reported to measure group differences. P‐values < 0.05 were considered statistically significant.

## 3 Results

### 3.1 Demographic Characteristics and Smoking Status

Most of the OLP and OLL patients were middle‐aged women (Table 1). OLL patients were older than OLP patients (*p* = 0.004). No significant difference was present in sex distribution or smoking habits between the study groups (Table 1). Information on smoking was missing for about a third of OLP and OLL patients and for almost half of control patients.

**TABLE 1:** Basic characteristics of OLP, OLL, combined OLP/OLL, and control groups.

	OLP patients (*n* = 205)			OLL patients (*n* = 96)		All OLP/OLL patients (*n* = 301)		Controls (*n* = 301)
		*p* OLP vs. OLL	*p* OLP vs. controls		*p* OLL vs. controls		*p* OLP/OLL vs. controls	
Mean age (range)	56 (15–90)	**0.004**	0.336	61 (17–91)	**0.021**	58 (17–91)	0.724	58 (16–88)
	*n* (%)			*n* (%)		*n* (%)		*n* (%)
Sex								
Female	146 (71)	0.108	0.387	59 (61)	0.310	205 (68)	0.861	205 (68)
Male	59 (29)			37 (39)		96 (32)		96 (32)
Smoking^a^								
Non‐smokers	96 (67)	0.734	0.149	43 (69)	0.739	139 (68)	0.151	99 (62)
Ex‐smokers	25 (17)			9 (15)		34 (17)		22 (14)
Smokers	22 (15)			10 (16)		32 (16)		38 (24)

*Note:* *p*‐values were calculated by ANOVA and Chi‐squared tests.

Abbreviations: OLL, oral lichenoid lesion; OLP, oral lichen planus.

^a^ Information on smoking was missing from 30.2% (*n* = 62) of OLP, 35.4% (*n* = 34) of OLL, and 47% (*n* = 142) of control patients.

### 3.2 Medical History and Comorbidities

Altogether 19.5% (*n* = 40) of OLP patients, 20.8% (*n* = 20) of OLL patients, and 15.9% (*n* = 48) of controls were generally healthy with no diagnosed medical conditions (Table 2). The use of regular medications is presented in Table 2.

**TABLE 2:** Thyroid gland diseases and other selected comorbidities in OLP, OLL, combined OLP/OLL, and control groups with numbers and percentages and estimated odds ratios (ORs) with 95% confidence intervals (CIs) and *p*‐values for bivariate associations adjusted for age and sex by logistic regression.

	OLP patients (*n* = 205)			OLL patients (*n* = 96)			All OLP/OLL patients (*n* = 301)			Controls (*n* = 301)
	*n* (%)	OR [95% CI]	*p*	*n* (%)	OR [95% CI]	*p*	*n* (%)	OR [95% CI]	*p*	*n* (%)
Healthy										
No	165 (80.5)	0.771 (0.484–1.228)	0.273	76 (79.2)	0.680 (0.378–1.224)	0.139	241 (80.1)	0.740 (0.486–1.128)	0.138	253 (84.1)
Yes	40 (19.5)	1		20 (20.8)	1		60 (19.9)	1		48 (15.9)
Medications										
Thyroxine medication	29 (14.1)	1.341 (0.779–2.308)	0.290	18 (18.8)	1.848 (0.970–3.522)	0.062	47 (15.6)	1.497 (0.922–2.432)	0.103	33 (11.0)
Any medication^a^	139 (67.8)	0.962 (0.652–1.442)	0.852	67 (69.8)	0.836 (0.492–1.416)	0.505	206 (68.4)	0.917 (0.637–1.318)	0.638	210 (69.8)
None	66 (32.2)	1		29 (30.2)	1		95 (31.6)	1		91 (30.2)
Thyroid gland disease										
Autoimmune HT	25 (12.2)	1.425 (0.793–2.562)	0.237	18 (18.8)	2.292 (1.175–4.47)	0.015	43 (14.3)	1.691 (1.005–2.845)	0.048	27 (9)
Any TD	34 (16.6)	1.376 (0.824–2.296)	0.222	19 (19.8)	1.707 (0.912–3.193)	0.094	53 (17.6)	1.478 (0.932–2.343)	0.096	38 (12.6)
None	171 (83.4)	1		77 (90.2)	1		248 (82.4)	1		263 (87.4)
Allergy										
Yes	74 (36.1)	1.872 (1.245–2.815)	0.003	25 (26.0)	1.410 (0.809–2.458)	0.226	99 (32.9)	1.726 (1.184–2.516)	0.005	69 (22.9)
No or unknown	131 (63.9)	1		71 (74.0)	1		202 (67.1)	1		232 (77.1)

Abbreviations: HT, hypothyroidism; OLL, oral lichenoid lesion; OLP, oral lichen planus; TD, thyroid disease.

^a^ Including all regular medications, including levothyroxine medication.

### 3.3 Thyroid Diseases

The prevalence of autoimmune HT (primary HT) was 12.2% (*n* = 25) in the OLP group, 18.8% (*n* = 18) in the OLL group, and 9% (*n* = 27) in the control group (Table 2). About 2% (*n* = 4) of OLP patients, 2% (*n* = 6) of controls, and none of the OLL patients had primary hypothyroidism due to a cause other than autoimmune HT (such as removal of adenoma or radiation for thyroid condition). Moreover, 2.4% (*n* = 5) of OLP, 1% (*n* = 1) of OLL, and 1.7% (*n* = 5) of the control participants had other thyroid diseases, such as hyperthyroidism or goiter without HT.

### 3.4 Allergies

Allergy is defined as an exaggerated response of the body's immune system to otherwise inert substances present in the environment (Dougherty et al. 2024). OLP patients were significantly more likely to have allergies than the control group (adjusted OR 1.87 [95% CI 1.245–2.815], *p* = 0.003) (Table 2). OLP patients were more likely to have allergies than OLL patients, as 36.1% (*n* = 74) of OLP and 26.0% (*n* = 25) of OLL patients had a history of allergy (Table 2). Two OLP patients (1%) were allergic to mercury, but the lesions persisted after amalgam fillings were replaced with composite fillings (Table S1). Five patients (5%) in the OLL group and no patients in the control group were allergic to mercury. A total of 35 OLP patients (17%), 8 OLL patients (8.3%), and 48 controls (16%) had allergy to some medicine. The most common hypersensitivities to medications were to penicillin (OLP 8.8% (*n* = 18), OLL 3.1% (*n* = 3), controls 4.7% (*n* = 14)) and sulfonamide (OLP 6.8% (*n* = 14), OLL 0% (*n* = 0), controls 2.3% (*n* = 7)). A complete list of reported allergies in the study groups is presented in Table S1.

### 3.5 Other Diseases and Reactions

Psoriasis was more common among OLP patients and coronary artery disease among OLL patients relative to controls without statistical significance (Table S2). Five OLP patients (2.4%) and 2 OLL patients (2%) had atopic eczema, and none of the controls had the disease. In addition, 5 OLP patients (2.4%), 1 OLL patient (1%), and no controls had celiac disease.

An increased risk for diabetes or hypertension was not observed among OLP or OLL patients (Table S2). Hepatitis C infection was not present in any of the OLP or OLL patients; 3 patients (1%) in the control group had hepatitis C infection.

Five percent (*n* = 5) of OLLs were contact reactions caused by amalgam fillings confirmed with patch testing. None of the OLL patients had GVHD or other specific diseases that may induce OLLs, or reliably identified lichenoid drug, cinnamon, or smokeless tobacco reactions. Therefore, in most of the OLL cases, the etiology remained unknown.

### 3.6 OLP and OLL Clinical Characteristics

Both OLP and OLL presented most often as at least two different clinical types (Table 3). Reticular and atrophic lesions were more common in OLP patients than in OLL patients (*p* < 0.001 and *p* = 0.003). However, plaque‐like lesions were significantly more common among OLL patients than among OLP patients (*p* = 0.01) (Table 3). Atrophic and erosive lesions without reticular lesions were rare; in the OLP group, there was one patient (0.5%) with only erosive and atrophic lesions and one patient (0.5%) with solely erosive lesions (Table 3). No significant difference was present in the number of patients in the groups by lesion type between OLP and OLL groups.

**TABLE 3:** Clinical features of oral lesions in OLP and OLL groups.

	OLP (*n* = 205)	OLL (*n* = 96)	*p* OLP vs. OLL
	*n* (%)	*n* (%)	
Clinical type^a^			
Reticular	198 (97)	70 (73)	< 0.001
Atrophic	146 (71)	54 (56)	0.003
Plaque‐like	50 (24)	38 (40)	0.011
Erosive	96 (47)	35 (36)	0.107
Papular	5 (2)	2 (2)	0.849
Bullous	0 (0)	1 (1)	0.143
Patient group by lesion type			
0 (reticular, papular, plaque)	53 (26)	34 (35)	0.123
1 (atrophic)	58 (28)	26 (27)	
2 (erosive, bullous)	94 (46)	36 (38)	
Patient group by location of lesions			
Buccal only	42 (21)	33 (34)	0.009
Buccal and gingival	28 (14)	7 (7)	0.108
Buccal and lingual	27 (13)	13 (14)	0.872
Buccal, gingival, and lingual	11 (5)	2 (2)	0.192
Gingival only	3 (2)	0 (0)	0.234
Lingual only	5 (2)	13 (14)	< 0.001
Other oral site^b^ only	3 (1)	5 (5)	0.06
Several sites^c^	86 (42)	23 (24)	0.005
LP elsewhere in the body	51 (25)	10 (10)	0.004

*Note:* *p*‐values were calculated by ANOVA and Chi‐squared tests.

Abbreviations: LP, lichen planus; OLL, oral lichenoid lesions; OLP, oral lichen planus.

^a^ If patient had several clinical types, all of them were counted.

^b^ Palatal mucosa, floor of mouth, labial mucosa, lip vermilion, alveolar ridge mucosa.

^c^ Excluding the previously mentioned groups.

LP was present elsewhere in the body in 24.9% (*n* = 51) of OLP patients (Table 3). A total of 20% (*n* = 42) of OLP patients had cutaneous LP, and 5.4% (7 women [4.8% of women], 4 men [6.7% of men]) had genital LP. One male in the OLP group had ocular LP in addition to genital and cutaneous LP. One woman with OLP had cutaneous LP, ocular LP, and LP in the scalp. One OLP patient had lichen unguis. Four women (2.7%) with OLP had genital and cutaneous LP. In the OLL group, 8.3% (*n* = 8) of patients had cutaneous LP or LL, and 2% (2 cases, both women) had genital LP or LL.

When OLP and OLL patients were divided according to the presence of HT, patients with HT more often belonged to group 2 (having erosive lesions) than patients without HT. A total of 58.6% (*n* = 17) of OLP patients with HT and 43.8% (*n* = 77) of OLP patients without HT had erosive lesions; the corresponding values in the OLL group were 44.4% (*n* = 8) and 35.9% (*n* = 28). However, these findings were not statistically significant (p‐value for OLP 0.305 and for OLL 0.777).

A total of 74% (*n* = 152) of OLP patients had at least two oral sites of involvement (Table 3). On the other hand, OLL patients were more likely to have lesions only in one location, as over 50% of the lesions were confined to one site, mainly buccal or lingual mucosa (*p*‐values 0.009 and < 0.001). Isolated lesions on the palatal mucosa, floor of the mouth, and the lip were uncommon. Among OLP and OLL patients with also extraoral LP, lesion distribution varied: 21.6% (11/51) of OLP and 10% (1/10) of OLL patients had buccal lesions only; 9.8% (*n* = 5) of OLP and 10% (*n* = 1) of OLL had both buccal and gingival lesions; and 7.8% (*n* = 4) of OLP and 10% (*n* = 1) of OLL presented with buccal, gingival, and lingual involvement. Isolated gingival or lingual lesions were less common, with 1.9% (*n* = 1) of OLP patients exhibiting gingival lesions only, and 1.9% (*n* = 1) of OLP and 20% (*n* = 2) of OLL patients showing only lingual involvement. Lesions on other isolated oral sites were seen in 1.9% (*n* = 1) of OLP cases. The majority, 45% (*n* = 23) of OLP and 50% (*n* = 5) of OLL patients, had multifocal involvement beyond the above categories.

## 4 Discussion

The previously observed positive association of oral lichenoid disease with thyroid diseases, especially autoimmune HT, was seen also here. It is noteworthy, however, that only the association between OLL and autoimmune HT reached statistical significance. In addition, OLP was associated with allergies. The findings also suggest that psoriasis and celiac disease may be more prevalent among OLP patients than among controls. An increased prevalence of hypertension, diabetes, or HCV infection in OLP and OLL patients was not observed.

OLP patients had significantly increased odds for allergies compared with the control group (*p* = 0.003). In particular, allergies to penicillin, sulfonamide, and pollen were more prevalent in OLP patients than in OLL or control patients (Table S1). Most of the OLP and OLL patients did not have documented allergy tests performed, which may influence the results. In Finland, pollen allergy, for instance, is not recommended to be tested with official allergy tests if the patient's symptoms are clear and the medication helps (Blomberg 2025). An increased prevalence of allergies in OLP patients has been previously reported in a different Finnish population (Siponen et al. 2010). In addition, in an Australian study, OLP patients had more often relevant reactions in patch tests to mercury‐related chemicals, amalgam, spearmint, and carvone than patients with cheilitis (*p* < 0.001) (Ting et al. 2023). Food allergens may trigger OLP flares in some patients, as shown in a case where specific dietary exposures exacerbated erosive OLP (Chen et al. 2016). Elimination of these allergens led to marked improvement and reduced need for systemic therapy, suggesting that immune responses to food antigens can contribute to OLP activity (Chen et al. 2016). However, not all studies have found an association between OLP and allergies (Kragelund et al. 2003). This discrepancy may be due to different genetic backgrounds and immune‐mediated mechanisms associated with OLP and allergy. Polymorphisms in genes like CTLA‐4 and IL‐4 are associated with both allergic and autoimmune diseases (Hossen et al. 2023; Michailidou et al. 2021; Shen et al. 2015; Yang et al. 2004), possibly explaining the different results.

The association of OLP with another immune‐mediated disease, autoimmune HT, has been investigated in several countries. Two meta‐analyses revealed a positive and significant association between OLP and HT (De Porras‐Carrique et al. 2022; Li et al. 2017). Studies of cutaneous LP, lichen planopilaris, and HT also suggest this association (Atanaskova Mesinkovska et al. 2014; Dreiher et al. 2009). In 2009, a Taiwanese study revealed that, among Chinese patients with OLP, 21% of patients had serum anti‐thyroglobulin autoantibodies and 24% had anti‐thyroid microsomal autoantibodies compared with 1.9% of healthy controls (Chang et al. 2009). An association between OLP and HT in a Northern Ostrobothnian population has previously been shown (estimated OR 2.39) (Siponen et al. 2010). In the present study on an Eastern Finnish population, OLL was associated with autoimmune HT (OR = 2.292, *p* = 0.015), but the association between OLP and autoimmune HT (OR = 1.425, *p* = 0.237) was, however, not statistically significant. The reasons underlying the contradictory results observed globally regarding the association between OLP and HT remain speculative (Aghbari et al. 2017; Kats et al. 2019; Lavaee and Majd 2016; Vucicevic Boras et al. 2014). The inconsistency among studies can be attributed to several factors, including methodological heterogeneity, variation in diagnostic criteria for both OLP and HT, and potential regional differences in the prevalence of the diseases. It could be hypothesized that within the same geographic regions, similar trends in disease frequency might emerge. However, even within identical geographical areas, conflicting results have been reported (Compilato et al. 2011; Lauritano et al. 2016; Lo Muzio et al. 2013) and now also within Finland. Both Finnish studies employed a comparable retrospective age‐ and sex‐matched case–control design, with diagnoses of OLP and OLL based on clinical and histopathological criteria. However, a difference lies in the diagnostic classifications applied: the earlier study adopted the criteria proposed by van der Meij and van der Waal (van der Meij and van der Waal 2003), whereas the present study employed the more recent criteria by the AAOMP (Cheng et al. 2016). The diagnostic criteria for OLP proposed by van der Meij and van der Waal emphasize the necessity of reticular lesions, together with a well‐defined, band‐like zone of cellular infiltration, composed predominantly of lymphocytes confined to the superficial lamina propria (van der Meij and van der Waal 2003). In contrast, the AAOMP criteria do not require the presence of reticular lesions but may exclude cases based on specific clinical features, such as lesions restricted to areas in direct contact with dental restorations (Cheng et al. 2016). Histopathologically, the AAOMP criteria permit either band‐like or patchy lymphocytic infiltration but require the presence of lymphocytic exocytosis and explicitly exclude cases demonstrating verrucous epithelial architecture (Cheng et al. 2016). Both the criteria proposed by van der Meij and van der Waal (van der Meij and van der Waal 2003) and those of the AAOMP (Cheng et al. 2016) classify cases that do not fully meet the diagnostic requirements for OLP as OLL. The AAOMP proposal, however, describes a more detailed subclassification of OLL, distinguishing entities with identifiable etiologic associations (Cheng et al. 2016). Owing to the differences in the diagnostic criteria for OLP between the two proposals, the designation of OLL may be applied to distinct subsets of patients.

Additional differences between the studies include geographical location within Finland (Western vs. Eastern), sample size, mean age, and sex distribution of the study cohorts. A nationwide study conducted in Finland found no significant regional variation in the prevalence of HT between university hospital districts (Virta and Eskelinen 2011), suggesting that geographic location alone may not explain the observed differences. However, Finland's demographic history has resulted in notable genetic stratification between the eastern and western regions. The genetic distance between Eastern and Western Finns is greater than that between several major European populations, such as the British and Germans (Jakkula et al. 2008). Eastern Finland is characterized by reduced haplotype diversity and longer runs of homozygosity (ROH), consistent with founder effects and prolonged genetic isolation (Kerminen et al. 2017; Salmela et al. 2008). Whether the genetic differences of the study populations between the two Finnish studies could explain the discrepancies seen in the results is unknown. However, a recent genome‐wide association study conducted in the Finnish population with 7679 LP cases identified nine genomic loci significantly associated with both LP and autoimmune HT (Reeve et al. 2024), suggesting a potential shared genetic basis for these comorbidities. Variations in diagnostic practices, population environmental exposures over time, and sample size differences between the earlier (Siponen et al. 2010) and current Finnish study may also influence the results of HT and OLP associations.

In the earlier Finnish study (Siponen et al. 2010), 71% of patients with OLP/OLL were women, with a mean age of 50 years. In the current study, women comprised 68% of the patient population, with a higher mean age of 58 years. Given that HT is significantly more prevalent among females, and that a Finnish study reported 84% of levothyroxine users were women (Virta and Eskelinen 2011), such demographic differences might be relevant. Numerous European and North American studies have consistently demonstrated that advancing age is a major risk factor for HT, particularly in its subclinical form (Canaris et al. 2000; Hollowell et al. 2002; Vanderpump et al. 1995). Nonetheless, since both Finnish studies included age‐ and sex‐matched controls, these minor differences in age and sex distribution are unlikely to explain the divergent findings.

A further complicating factor in the results might be the high proportion of undiagnosed HT in the general population. Based on a European systematic review, the prevalence of subclinical HT is 4.11%, and HT often goes undiagnosed (Mendes et al. 2019). This lack of systematic thyroid screening also in Finnish patients could mean that some patients might have asymptomatic HT, potentially influencing the results. However, a Chinese prospective study demonstrating a significant association between both OLP and OLL and autoimmune HT also included systematic thyroid function testing and ultrasonography (Zhou et al. 2018).

In the Northern Ostrobothnian study, the diagnoses of OLP and OLL were given by both general dentists and specialists. Here, the diagnoses were given only by specialists, which may have resulted in more accurate diagnoses. By following the strict set of criteria for OLP proposed by AAOMP (Cheng et al. 2016), the diagnosis of OLL is rendered by exclusion and not by inclusion. It is recognized that this proposed guideline may exclude some OLP cases (Cheng et al. 2016), with a diagnosis of OLL given instead, possibly affecting the prevalence of HT in the groups.

The mechanism between the association of OLP, OLL, and thyroid diseases remains unclear. This study supports the results of previous reports showing that thyroid diseases, especially HT and OLP and OLL, may have a common factor affecting their development. A molecular mimicry hypothesis has been proposed for both LP and HT, in which structural similarity between microbial antigens and human autoantigens can turn a defensive immune reaction into an autoimmune reaction in genetically predisposed individuals (Guarneri et al. 2017). Several authors have suggested that there may be a common genetic susceptibility for LP and thyroid disease by analyzing the links between specific alleles of the *HLA* genes in these diseases (Guarneri et al. 2017). A recently published broad genome‐wide association study supports this, indicating variation at nine genomic loci that is strongly associated with both LP and autoimmune HT (Reeve et al. 2024).

Altogether 12.2% of OLP patients had autoimmune HT, which lies between the values observed in previous studies (Lo Muzio et al. 2013; Siponen et al. 2010). In the earlier Finnish study (Siponen et al. 2010) where the association between OLP and HT was significant, 10% of OLP patients had HT, which is less than in the current study. Here 9% of the control group had autoimmune HT, which is more than in the earlier Finnish study (5%) (Siponen et al. 2010) and in Finland in general, potentially explaining why the association between OLP and autoimmune hypothyroidism was not significant in the current study. According to the Social Insurance Institution of Finland, which maintains a record of patients with prescribed medications, 1.5% of the Finnish population had a medication for HT at the end of the year 2020 (Kansaneläkelaitos 2020). However, this figure includes persons of all ages in the Finnish population; the percentage of people with HT is higher in older age groups. The difference between the two control groups may be partly explained by the higher mean age of the control group in the present study (57.6 vs. 50 years in the other study). The controls also likely had more health problems, as they were treated in a university hospital.

Almost 19% of OLL patients had autoimmune HT, which is a high proportion and a statistically significant finding. The same association between OLL and autoimmune HT was seen in a different genetic population in China, where the prevalence of thyroid diseases is high (Zhou et al. 2018). In an Italian study, which used the diagnostic criteria proposed by van der Meij et al. (van der Meij and van der Waal 2003), thyroid diseases were associated with OLL, but HT studied separately did not reach statistical significance (Piloni et al. 2024). Although the earlier Finnish study (Siponen et al. 2010) did not report a significant association between OLL and HT, likely due to the relatively small sample size (*n* = 70), a higher proportion of OLL patients were diagnosed with HT than controls (9% vs. 5%), suggesting a potential association. In the present study, the larger sample size (*n* = 96) may have increased the statistical power, thereby facilitating the detection of a significant relationship between these comorbidities. A meta‐analysis suggested that routine screening for thyroid disease might be beneficial for OLP patients (Li et al. 2017). Based on our findings, this could be worthy of consideration also among OLL patients since subclinical HT is associated with increased mortality (Inoue et al. 2020).

Compared with the idiopathic nature of OLP, OLLs may have an identifiable inciting factor. Certain drugs (e.g., antihypertensive, antimalarial, non‐steroidal anti‐inflammatory, and antimicrobial), dental restorative materials, GVHS, and other factors (e.g., dental tartar) can be involved in the pathogenesis of OLL (Binnie et al. 2024; Muller 2017). In the present study, in most cases, the aetiological factor for OLL remained unclear. It has been reported that in a considerable proportion of OLL cases, no definitive aetiological factor can be identified, even after comprehensive clinical examination and detailed medical history assessment, leaving the underlying cause of the lesions unknown (Xue et al. 2005). The application of strict diagnostic criteria for OLP, as outlined by AAOMP (Cheng et al. 2016), may partially account for this, as atypical presentations of OLP are classified as OLL. Additionally, it is possible that some OLL cases represent drug‐induced reactions; however, the extended latency period, averaging 16 weeks, poses a diagnostic challenge (Maul et al. 2023). Confirmation of the diagnosis of an OLDR can be difficult, and the incidence is unknown since empirical withdrawal of the suspected drug may be complicated (Al‐Hashimi et al. 2007).

In the present study, it was observed that OLP and OLL patients with HT more often had erosive OLP or OLL clinical types than patients without HT. In a Taiwanese study, 46.4% of patients with desquamative gingivitis and erosive OLP were positive for anti‐thyroglobulin antibodies and 45.1% were positive for anti‐thyroid microsomal antibodies. The corresponding proportions among patients with erosive OLP but not gingivitis were 27.5% and 30.3%, respectively (Chang et al. 2017). In addition, another study revealed that severe erosive OLP was a significant risk factor for anti‐thyroglobulin/anti‐thyroid microsomal autoantibody positivity in OLP patients (Chang et al. 2009). This might imply that thyroid disease affects the clinical features of OLP. Even though the association of HT and the erosive clinical type of OLP or OLL lesions in the present study was not statistically significant, it appears that HT or use of thyroxine may affect the clinical features. In this study, OLL was more strongly associated with HT than OLP, and it is possible that HT may alter the clinical features of OLP towards less typical presentation, resulting in an OLL diagnosis. However, it is noteworthy that reports on thyroxine medication causing lichenoid drug reactions are rare. As the sample size in this study is small, larger studies are needed to elucidate these findings.

Strengths of this study were that all patient data in both the study groups and the control group were gathered and the diagnosis of OLP or OLL was made by a specialist combining the clinical and histopathological presentations. In addition, the study groups and the control group comprised patients who had visited the same clinic during the 21‐year period from 1996 to 2017.

However, it is not possible to control all variables in a retrospective study. Some information in the medical charts may be missing or inaccurate, as patients and doctors report information differently. However, as the patients visited a university hospital, the medical records are usually gathered accurately. In addition, the sample size in this study is rather small, which might affect the results. Blinding was not feasible in this study, as the first author (M.V.) was responsible for both data collection and analysis. The diagnoses of OLP and OLL were made by a single investigator, which introduces a potential risk of observer bias. Nevertheless, the diagnostic assessments were conducted using the strict criteria described previously, and data collection and analysis were carried out by a clinical specialist (M.V.) who was instructed by the study supervisors to apply the diagnostic criteria consistently and to minimize subjective interpretation. Moreover, the authors declare no conflicts of interest, reducing the likelihood of bias influencing the findings.

Both psoriasis and celiac disease were observed more frequently in patients with OLP than in controls; however, these associations did not reach statistical significance, likely reflecting the limited sample size and wide confidence intervals. Recent evidence has strengthened the plausibility of these associations. A case–control study was the first to demonstrate a significant link between OLP and psoriasis (OR 3.13; 95% CI 1.20–9.68) (Rodriguez‐Fonseca et al. 2024), a finding subsequently confirmed in a population‐based Finnish study, which also proposed a shared genetic background (Reeve et al. 2024). Specifically, the variant *TNRC18*, previously linked to LP (Reeve et al. 2024), was also linked to psoriasis (Kurki et al. 2022). In addition, the same Finnish study reported a significant association between OLP and celiac disease (Reeve et al. 2024). Similarly, another investigation found that celiac disease was significantly more prevalent among OLP patients than among healthy controls, with the OLP group demonstrating higher frequencies of HLA‐DQ2 and HLA‐DQ8 alleles, consistent with a potential shared genetic predisposition (Cigic et al. 2015).

In conclusion, OLL was significantly associated with HT, and OLP was associated with allergies. Autoimmune HT was more common among OLP patients than among controls, but the association was not statistically significant. Future studies with larger sample sizes are needed to examine the possible association between OLP, OLL, and other comorbidities, especially psoriasis and celiac disease. In addition, further investigations may reveal whether HT influences the clinical features of OLP and OLL.

## Author Contributions

**Mari Vehviläinen:** writing – original draft, formal analysis, investigation, methodology, data curation, validation, funding acquisition. **Tuomas Selander:** writing – review and editing, methodology, validation, supervision. **Tuula Salo:** writing – review and editing, supervision, methodology, validation. **Maria Siponen:** conceptualization, supervision, resources, project administration, writing – review and editing, methodology, validation.

## Ethics Statement

The study protocol was approved by the Ethical Committee of the Northern Ostrobothnian Hospital District (7449/06.01.03.01/2013).

## Conflicts of Interest

The authors declare no conflicts of interest.

## Supporting information


**Table S1:** odi70117‐sup‐0001‐TableS1.docx.

**Table S2:** odi70117‐sup‐0002‐TableS2.docx.

## Data Availability

The data that support the findings of this study are available on request from the corresponding author. The data are not publicly available due to privacy or ethical restrictions.
