# Supplementary material for: Systemic Disease Associations of Oral Lichenoid Disease: A Retrospective Case–Control Study
Source: Oral Dis. 2025 Oct 9;32(6):1600–10. doi: 10.1111/odi.70117 (PMC13457676; doi:10.1111/odi.70117)
Supplement: Supplementary file 1 [file ODI-32-1600-s001.docx]

|  | OLP patients (n=205)  n (%) OR [95% CI] p-value | | | OLL patients (n=96)  n (%) OR [95% CI] p-value | | | All OLP/OLL patients (n=301)  n (%) OR [95% CI] p-value | | | Controls (n=301)  n (%) |
| --- | --- | --- | --- | --- | --- | --- | --- | --- | --- | --- |
| Psoriasis  Yes  No | 5 (2.4)  200 (97.6) | 3.724 (0.714-19.429)  1 | 0.119 | 1 (1)  95 (99) | 1.624 (0.144-18.277)  1 | 0.695 | 6 (2)  295 (98) | 3.065 (0.613-15.319)  1 | 0.172 | 2 (0.7)  299 (99.3) |
| Diabetes  Yes  No | 18 (8.8)  187 (91.2) | 1.383 (0.711-2.688)  1 | 0.339 | 6 (6.3)  90 (93.8) | 0.754 (0.291-1.957)  1 | 0.562 | 24 (8)  277 (92) | 1.149 (0.622-2.123)  1 | 0.657 | 21 (7)  280 (93) |
| Hypertension  Yes  No | 64 (31.2)  141 (68.8) | 1.322 (0.883-2.012)  1 | 0.172 | 28 (29.2)  68 (70.8) | 0.844 (0.490-1.452)  1 | 0.540 | 92 (30.6)  209 (69.4) | 1.150 (0.793-1.664)  1 | 0.462 | 83 (27.6)  218 (72.4) |
| Coronary artery disease  Yes  No | 14 (6.8)  191 (93.2) | 1.135 (0.548-2.350)  1 | 0.734 | 14 (14.6)  82 (85.4) | 1.721 (0.801-3.695)  1 | 0.164 | 28 (9.3)  273 (90.7) | 1.357 (0.733-2.513)  1 | 0.332 | 21 (7)  280 (93) |
| Hypercholesterolaemia  Yes  No | 30 (14.6)  175 (85.4) | 1.074 (0.639-1.806)  1 | 0.786 | 16 (16.7)  80 (83.3) | 0.884 (0.459-1.702)  1 | 0.713 | 46 (15.3)  255 (84.7) | 1.004 (0.632-1.594)  1 | 0.988 | 45 (15)  256 (85) |

**Supplementary Table 2**. Other selected comorbidities in OLP, OLL, combined OLP/OLL, and control groups with numbers and percentages and estimated odds ratios (ORs) with 95% confidence intervals (CIs) and p-values for bivariate associations adjusted for age and sex by logistic regression.

OLP, oral lichen planus; OLL, oral lichenoid lesion.
