# Supplementary material for: Systemic Disease Associations of Oral Lichenoid Disease: A Retrospective Case–Control Study
Source: Oral Dis. 2025 Oct 9;32(6):1600–10. doi: 10.1111/odi.70117 (PMC13457676; doi:10.1111/odi.70117)
Supplement: Supplementary file 2 [file ODI-32-1600-s002.docx]

**Supplementary Table 1.** Reported allergies in oral lichen planus (OLP), oral lichenoid lesion (OLL), and control groups.

| Allergen | OLP patients (n=205) | OLL patients (n=96) | Controls (n=301) |
| --- | --- | --- | --- |
|  | n (%) | n (%) | n (%) |
| *Medications* |  |  |  |
| Penicillin | 18 (8.8) | 3 (3.1) | 14 (4.7) |
| Sulfonamide | 14 (6.8) | 0 (0) | 7 (2.3) |
| Iodine (iodinated radiological contrast media or contact allergy to povodine-ionide) | 0 (0) | 3 (3.1) | 2 (0.7) |
| Salicylic acid | 1 (0.5) | 0 (0) | 4 (1.3) |
| Doxycycline | 2 (1) | 0 (0) | 2 (0.7) |
| Cephalosporin | 1 (0.5) | 0 (0) | 3 (1) |
| Trimethoprim | 1 (0.5) | 1 (1) | 1 (0.3) |
| Tacrolimus | 1 (0.5) | 0 (0) | 1 (0.3) |
| Paracetamol | 1 (0.5) | 0 (0) | 1 (0.3) |
| Miconazole | 0 (0) | 1 (1) | 1 (0.3) |
| Neomycin | 1 (0.5) | 0 (0) | 0 (0) |
| Erythromycin | 1 (0.5) | 0 (0) | 0 (0) |
| Terbinafine | 1 (0.5) | 0 (0) | 0 (0) |
| Hydrocortisone | 1 (0.5) | 0 (0) | 0 (0) |
| Naproxen | 1 (0.5) | 0 (0) | 0 (0) |
| Tranexamic acid | 0 (0) | 0 (0) | 1 (0.3) |
| Tetracycline | 0 (0) | 0 (0) | 1 (0.3) |
| Clindamycin | 0 (0) | 0 (0) | 1 (0.3) |
| Metronidazole | 0 (0) | 0 (0) | 1 (0.3) |
| Phenytoin | 0 (0) | 0 (0) | 1 (0.3) |
| Carbamazepine | 0 (0) | 0 (0) | 1 (0.3) |
| Paracetamol+codeine | 0 (0) | 1 (1) | 0 (0) |
| *Others* |  |  |  |
| Pollen | 21 (10) | 4 (4.2) | 4 (1.3) |
| Food ingredients | 8 (3.9) | 4 (4.2) | 3 (1) |
| Nickel | 8 (3.9) | 3 (3.1) | 5 (1.7) |
| Animal dust | 7 (3.4) | 3 (3.1) | 3 (1) |
| Mercury | 2 (1) | 5 (5) | 0 (0) |
| Perfumes | 3 (1.4) | 3 (3.1) | 0 (0) |
| Latex | 2 (1) | 1 (1) | 2 (0.7) |
| Mold | 2 (1) | 0 (0) | 1 (0.3) |
| Gadolinium | 0 (0) | 1 (1) | 0 (0) |
| Palladium chloride | 1 (0.5) | 0 (0) | 0 (0) |
| Methyl paraben | 1 (0.5) | 0 (0) | 0 (0) |
| Chrome | 0 (0) | 0 (0) | 1 (0.3) |
| Cobalt | 0 (0) | 0 (0) | 1 (0.3) |
| Thimerosal | 0 (0) | 0 (0) | 1 (0.3) |
|  |  |  |  |
